# New Intraoperative Imaging Tools and Image-Guided Surgery in Gastric Cancer Surgery

**DOI:** 10.3390/diagnostics12020507

**Published:** 2022-02-16

**Authors:** Luise Knospe, Ines Gockel, Boris Jansen-Winkeln, René Thieme, Stefan Niebisch, Yusef Moulla, Sigmar Stelzner, Orestis Lyros, Michele Diana, Jacques Marescaux, Claire Chalopin, Hannes Köhler, Annekatrin Pfahl, Marianne Maktabi, Ji-Hyeon Park, Han-Kwang Yang

**Affiliations:** 1 Department of Visceral, Transplant, Thoracic and Vascular Surgery, University Hospital of Leipzig AöR, 04103 Leipzig, Germany; marie.knospe@medizin.uni-leipzig.de (L.K.); boris.jansen-winkeln@sanktgeorg.de (B.J.-W.); rene.thieme@medizin.uni-leipzig.de (R.T.); stefan.niebisch@medizin.uni-leipzig.de (S.N.); yusef.moulla@medizin.uni-leipzig.de (Y.M.); sigmar.stelzner@medizin.uni-leipzig.de (S.S.); orestis.lyros@medizin.uni-leipzig.de (O.L.); 2 Department of General, Visceral and Oncological Surgery, St. Georg Hospital, 04129 Leipzig, Germany; 3 Institute for Research against Digestive Cancer (IRCAD), 67091 Strasbourg, France; michele.diana@ircad.fr (M.D.); jacques.marescaux@ircad.fr (J.M.); 4 ICUBE Laboratory, Photonics Instrumentation for Health, University of Strasbourg, 67400 Strasbourg, France; 5 Department of General, Digestive, and Endocrine Surgery, University Hospital of Strasbourg, 67091 Strasbourg, France; 6 Innovation Center Computer Assisted Surgery (ICCAS), Leipzig University, 04103 Leipzig, Germany; claire.chalopin@iccas.de (C.C.); hannes.koehler@medizin.uni-leipzig.de (H.K.); annekatrin.pfahl@medizin.uni-leipzig.de (A.P.); marianne.maktabi@medizin.uni-leipzig.de (M.M.); 7 Department of Surgery, Seoul National University Hospital, Seoul 03080, Korea; pjhaaa1220@gmail.com (J.-H.P.); hkyang@snu.ac.kr (H.-K.Y.)

**Keywords:** innovative intraoperative imaging, image-guided surgery, indocyanine green (ICG), fluorescence imaging (FI), hyperspectral imaging (HSI)

## Abstract

Innovations and new advancements in intraoperative real-time imaging have gained significant importance in the field of gastric cancer surgery in the recent past. Currently, the most promising procedures include indocyanine green fluorescence imaging (ICG-FI) and hyperspectral imaging or multispectral imaging (HSI, MSI). ICG-FI is utilized in a broad range of clinical applications, e.g., assessment of perfusion or lymphatic drainage, and additional implementations are currently investigated. HSI is still in the experimental phase and its value and clinical relevance require further evaluation, but initial studies have shown a successful application in perfusion assessment, and prospects concerning non-invasive tissue and tumor classification are promising. The application of machine learning and artificial intelligence technologies might enable an automatic evaluation of the acquired image data in the future. Both methods facilitate the accurate visualization of tissue characteristics that are initially indistinguishable for the human eye. By aiding surgeons in optimizing the surgical procedure, image-guided surgery can contribute to the oncologic safety and reduction of complications in gastric cancer surgery and recent advances hold promise for the application of HSI in intraoperative tissue diagnostics.

## 1. Introduction

Further to laparoscopic and robotic technologies, innovations and new advancements in intraoperative real-time imaging have gained significant importance in the recent past in the field of gastric cancer surgery. Image-guided surgery opens up new possibilities for the intraoperative visualization of important surgical parameters, e.g., tissue perfusion and lymphatic drainage, and thus provides valuable decision support in the course of surgical intervention. The aim of the application of the described invasive or non-invasive imaging procedures and the associated information gain is to optimize the course of surgery, reduce postoperative complications, and thus to increase patient safety. Tissue characteristics or tissue conditions that are initially indistinguishable for the human eye can be visualized with high accuracy and different methods of quantification have already been established in several areas. The most common and well-established imaging modality in gastric cancer surgery is indocyanine green fluorescence imaging (ICG-FI), which is already applied in a broad range of clinical applications. The procedure has shown promising results in securing the completeness of lymph-node dissection, facilitating surgical dissection, and visualizing anastomotic perfusion and might be implemented in further areas of application in the future. Another highly promising technique is hyperspectral (or multispectral) imaging (HSI or MSI), as it does not require the application of a contrast agent and provides further information on the condition and composition of the examined tissue, on top of different perfusion parameters. The procedures are still in an experimental phase, but preliminary studies have shown a successful application in perfusion assessment of gastrointestinal anastomoses and promising prospects concerning tissue and tumor classification [1]. Future developments are aimed at the integration of artificial intelligence (AI)—in particular of algorithms—with the aid of deep learning or machine learning procedures into the further evaluation of the acquired image data. This would constitute an important advancement in enabling a more rapid and accurate intraoperative diagnosis and aid surgeons in optimizing the surgical procedure.

## 2. Perfusion Assessment with Indocyanine Green (ICG) Fluorescence Imaging (FI)

Indocyanine green is a fluorescent dye with an excitation wavelength of approximately 800 nm. It can be administered intravenously or injected into the tissue at the target site, depending on the investigated parameters. Illumination at the specific excitation wavelength leads to the emission of light with longer wavelengths (as of 810 mm), which can be detected by near-infrared (NIR) cameras. In addition to the detection of sentinel lymph nodes and initial investigations on the identification of peritoneal and liver metastases, the method is currently used primarily for perfusion analysis in a variety of surgical interventions.

**Figure 1:**
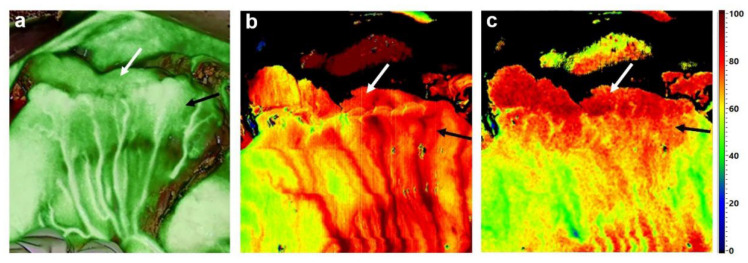
Perfusion assessment by ICG-FI and HSI during oncologic subtotal (7/8) D2-gastrectomy. After completion of the gastrojejunal anastomosis by manual suture, ICG-FI and HSI are performed to ensure sufficient perfusion of the gastric stump (white arrows) and the jejunal loop (black arrows). ICG-FI evidenced very well maintained blood flow in both segments, represented by a high color intensity of the calculated overlay (**a**). Both HSI index parameters, Near-infrared perfusion index (NIR-PI) (**b**) and tissue oxygenation (StO2) (**c**), also verified good perfusion.

While comprehensive and convincing data (prospective-randomized studies and meta-analyses) on the reduction of anastomotic complications with the application of indocyanine green fluorescence imaging (ICG-FI) have been published [2,3,4], there is a paucity of results of comparable studies on gastric cancer surgery. In the course of the described surgical interventions, ICG-FI measurements are performed in particular after subtotal (e.g., 4/5 or 7/8) gastric resection to evaluate gastric stump perfusion (Figure 1a). Kim et al. impressively demonstrated the application of ICG for real-time vessel navigation in the course of robotic or laparoscopic subtotal gastric resection or gastrectomy [5]. Recent studies from East Asia suggest that fluorescence-based perfusion assessment can be safely accomplished and might represent a valuable tool for the clinical prediction of anastomotic leakage [6,7]. In a retrospective study by Mori et al., multivariate analyses showed the time difference (TD = subtraction of the time until ICG fluorescence was visible at one end of the anastomosis from the time until fluorescence was depicted at the other end) to be an independent predictor for anastomotic leakage (odds ratio (OR): 35.36; 95% confidence interval (CI): 1.49–839.92; *p* = 0.027) [6]. However, the number of patients and rate of anastomotic insufficiency reported for the described study was relatively low (4/100 patients) and the clinical relevance of the parameter “time difference” (TD) therefore needs to be re-evaluated in a larger cohort of patients.

## 3. Intraoperative Guidance and Tumor Localization by Fluorescent Marking Clips

With the increasing implementation and associated benefits of minimally invasive procedures in gastric cancer surgery, certain disadvantages have also become evident and different approaches to overcoming these limitations are currently being evaluated. One of the major restrictions of totally laparoscopic or robotic techniques is that the surgeon is not able to palpate intraabdominal tissue, which inhibits the determination of the exact tumor localization. This might be particularly important in cases of early gastric cancer, where total gastrectomy might be averted in favor of a stomach-preserving procedure [8]. Park et al., recently reported interesting results regarding the effect of intraoperative gastroscopy (IOG) on the adequacy of surgical resection margins, as well as the prevention of unnecessary total gastrectomy. The authors compared the results of 1084 gastric cancer patients, consisting of three subgroups: extracorporeal gastrectomy, intracorporeal gastrectomy without the application of IOG, and intracorporeal gastrectomy using simultaneous gastroscopic and laparoscopic view to determine the most advantageous resection margin based on endoscopically applied marking clips. The results of this study highlight the importance of exact intraoperative tumor localization, as the intracorporeal IOG-guided group showed increased margin safety and found IOG to be an independent factor that prevented total gastrectomy [8].

A promising new approach to visualize the exact tumor localization without the need for haptic feedback or IOG might be the endoscopic application of fluorescent marking clips equipped with resin-conjugated ICG [9]. Namikawa et al. have reported promising first results in cases of gastric cancer, as well as other gastrointestinal tumors. The fluorescence signal of the preoperatively applied marking clips could be visualized on the serosal surface in 6 out of 8 cases and clearly indicated the intended position [10]. Even though further studies are needed to determine the reliability and accuracy of this method, the advantages of a non-haptic, non-invasive intraoperative detection of the tumor site, without the need for IOG or other devices, like intraoperative ultrasound, is highly promising.

## 4. Lymph Node Mapping with ICG-FI in Gastric Carcinoma

NIR-fluorescence-based lymphatic imaging has also attracted increasing interest in the field of sentinel node navigation and lymph node mapping in gastric carcinoma. The results of a large prospective-randomized study published by Chen et al. presented convincing evidence of the safety and efficacy of ICG-supported lymph node dissection in total gastrectomy with D2-lymphadenectomy for gastric cancer [11]. The authors reported the endoscopic peritumoral injection of ICG into the submucosa one day preoperatively. The mean number of dissected lymph nodes in the ICG group ranged at 50.5 lymph nodes (±15.9 SD) vs. 42.0 (±10.3 SD), and was significantly larger (*p* < 0.001) in the non-ICG group at comparable reconvalescence and similar complication rates in both cohorts. The authors concluded that ICG-FI could be applied for routine lymph node mapping during laparoscopic gastrectomy and especially during total gastrectomy.

Similar results for lymph node mapping during gastric cancer surgery have recently been reported by Cianci et al. [12]. The described study aimed to investigate whether ICG fluorescence lymphography has the potential to improve intraoperative visualization of lymph nodes in a matched cohort study during robotic gastrectomy for gastric cancer. The mean total number of harvested lymph nodes was significantly higher in the ICG than in the control group (50.8 vs. 40.1; *p* = 0.03). In the ICG cohort, 23 of 37 patients had metastatic lymph nodes. The accuracy, sensitivity, and specificity of ICG-FI for metastatic lymph nodes were 62.2%, 52.6%, and 63.0%, respectively. The authors concluded that fluorescence imaging with ICG might enable additional node detection during robotic surgery for gastric cancer. However, ICG fluorescence lymphography failed to show good selectivity for metastatic lymph nodes [12]. In interpreting studies reporting increasing harvest of lymph nodes by ICG-FI, we have to pay attention that D2-lymphadenectomy is defined by anatomic landmarks. If the surgeon fulfils proper dissection, the total lymph node harvest would not be affected by the addition of ICG-FI. The total number of harvested lymph nodes is, thus, also influenced by pathologists’ procedures or efforts. Therefore, it is necessary to carefully compare the ICG-FI group vs. non-ICG-FI group. It should also be considered that recent studies observed a decline in sensitivity for detecting all metastatic stations in advanced tumor stages [13,14]. Park et al., examined the applicability of ICG-FI in the mapping of perigastric lymphatic networks in cases of advanced gastric cancer, documenting the topography and ICG-staining of each lymph node and lymph node station and comparing it with corresponding pathological findings. Particularly interesting was that ICG staining missed a high number of lymph nodes, staining only 40% of the confirmed metastatic lymph nodes and 75% of the affected lymph node stations. Though further investigations are necessary, it should be taken into account that ICG-staining might not be considered safe to reduce the extent of lymph node dissection in advanced gastric cancer [13].

Kim et al., emphasized the benefit of ICG-FI to ensure the completeness of infrapyloric lymph node dissection, which is considered the most challenging step in radical lymphadenectomy in gastric cancer surgery. The authors concluded that ICG-FI may provide additional node detection and, thus, serve as a complementary tool for those surgeons who have limited experience with gastric cancer lymph node dissection or low volume centers to confirm the completeness of the lymphadenectomy [15]. A recent study evaluated the possible benefit of ICG-FI for secure infrapyloric lymph node dissection in laparoscopic distal gastrectomy. The technique was associated with a significantly lower rate of bleeding events and shorter time required from omentectomy to right gastro-epiploic vein exposure in the ICG group, resulting from enhanced visual contrast between the mesocolon and the mesogastric tissue provided by ICG-FI. This facilitated tracing of the dissectible layer and enabled clear identification of the avascular plane as well as the distinction of blood vessels from surrounding lymphatic structures. According to these findings, ICG-enhanced guidance facilitates safe and complete infrapyloric lymph node dissection in terms of precision oncology with shorter operation time and less blood loss [16,17].

It is important to consider that the results of ICG-FI for lymph node detection might also depend on the timing and dosage of ICG-administration, as well as the method of application. A recent study by Chen et al. found the approaches of preoperative submucosal and intraoperative subserosal injection to be comparable [18], but stronger evidence from larger, randomized studies will be needed to decide on an optimal protocol for a standardized ICG-FI procedure.

## 5. Detection of Micrometastases in the Liver and Peritoneal Carcinomatosis

In the future, the application of ICG might also be extended to aid the detection of liver micrometastases in the course of staging laparoscopy in gastric cancer and cancers of the esophagogastric junction (Siewert types II/III EGJ (esophago-gastric junction) tumors). Although these metastases are frequently not detected with conventional imaging techniques, e.g., computed tomography (CT), they play an important role in determining the strategy for the treatment algorithm. The ICG-guided detection of liver metastases has not yet been sufficiently investigated in cases of gastric or EGJ-cancer but revealed promising results in other entities, which encourage further research. Van der Vorst et al., studied the application of ICG-FI during hepatic metastasectomy for colorectal liver metastases. The authors did not only find a common pattern in the entrapment of ICG in metastatic lesions, which resulted in a fluorescent rim around the tumor, but they also identified additional, otherwise occult liver metastases in 12.5% (95% CI 5.0–26.6) of the patients. The described additional lesions were not identified with the use of any of the conventional imaging modalities (i.e., preoperative CT scan, intraoperative ultrasound (IOUS), intraoperative visualization by a surgeon’s eye, and intraoperative palpation) [19]. Similar results were obtained in a study by Peloso et al., who applied a combination of ICG-FI and IOUS to detect hepatic metastases rather than using IOUS and CT alone. Differences among the three imaging methods were most apparent in the detection of lesions ≤3 mm in diameter. The combined use of IOUS + ICG-FI was found to be most successful, detecting 29 lesions in contrast to 15 lesions found by IOUS alone (*p* = 0.0328) and nine lesions detected by preoperative CT alone (*p* < 0.0001). The authors concluded that the use of concomitant IOUS and ICG-FI may increase the accuracy of intraoperative exploration and dramatically improve the chances of complete tumor eradication [20].

Further promising utilization of ICG-FI is the intraoperative detection of peritoneal carcinomatosis. Baiocchi et al., recently conducted a systematic review, evaluating the possible role of ICG-FI in detecting peritoneal carcinomatosis in the course of the surgical treatment of abdominal malignancies. Although a small number of studies have reported the successful application of ICG-FI to support the completeness of cytoreductive surgery in patients with known peritoneal carcinomatosis and a limited number of studies have described the detection of previously unknown peritoneal dissemination, the application of ICG-FI has not yet been extensively investigated in laparoscopic settings and its applicability in cases of gastric cancer or cancer of the EGJ has yet to be confirmed. Even though only limited knowledge is currently available, it serves as an indication that ICG-FI might make a valuable contribution to the diagnostic algorithm in the future. A significant increase in the current average sensitivity (88.2%) and specificity (77.8%) rates may be anticipated with the application of more tailored approaches, as well as following the exclusion of subgroups with reduced predictive accuracy, e.g., mucinous colorectal cancers, specific neoadjuvant therapies, and with the availability of more prospectively and systematically acquired clinical data [21].

## 6. Hyperspectral Imaging (HSI)

Hyperspectral imaging (HSI) is a relatively new method used in image-guided and precision surgery. It has shown promising results in perfusion analysis and recognition/characterization of tissues/tumors [22,23,24,25,26,27]. Moreover, HSI has been successfully applied to distinguish between normal, precancerous, and cancerous tissue [28], has spectral-spatial classification properties for non-invasive cancer [29,30], and facilitates residual tumor identification during surgery [22]. Through the assessment of different reflectance properties, HSI permits a comprehensive evaluation of physiologic tissue parameters, such as perfusion, oxygenation, and water content [31,32,33]. To date, HSI has therefore been applied predominantly in wound imaging and wound management in plastic surgery transplants, vascular surgery, chronic wounds, and burn injuries [34,35,36,37]. The applicability of the system for the assessment of tissue parameters of gastrointestinal anastomoses has recently been demonstrated for the first time in visceral surgery by our group in vivo [26,33,38,39,40]. Preliminary studies also indicate the applicability of HSI in identifying exact perfusion-based resection planes in anatomic liver resection, as well as the suitability of the method to discriminate tissue perfusion in acute mesenteric ischemia and assess the viability of kidney allografts [41,42,43].

**Figure 2:**
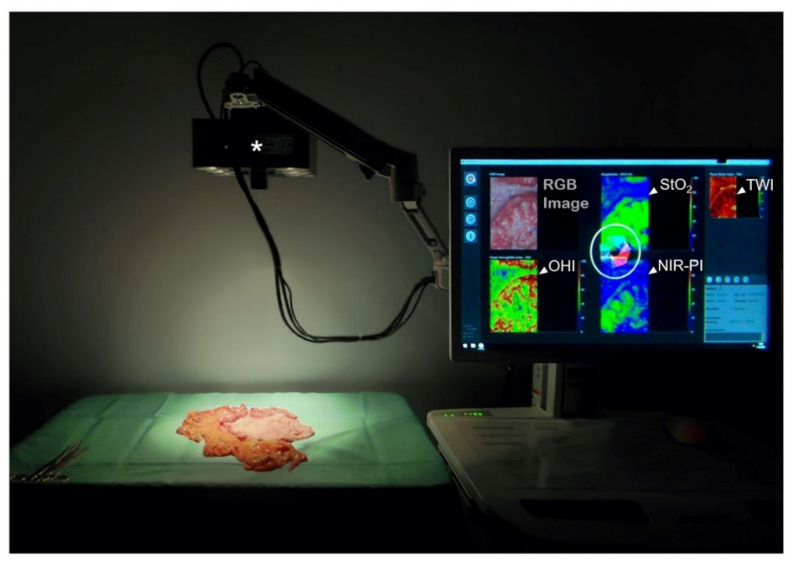
Acquisition of hyperspectral images with the TIVITA^®^ system. The HSI camera (*) is positioned at a distance of 50 cm above the object of interest. Six integrated halogen spots illuminate the field of view during the measurement process. After internal processing, an RGB image and physiological tissue parameters, visualized as false-color images (◅), are displayed on a connected monitor within 12 s from the initiation of the measurement.

HSI is a non-invasive, contact-free method that does not require the application of a contrast medium. At our three centers, hyperspectral images are acquired with the TIVITA^®^ Tissue System (Diaspective Vision GmbH, Am Salzhaff, Germany). The described HSI-camera is equipped with a push broom scanner, supplying images with a high spectral resolution (5 nm) in the visible and near-infrared range (500–1000 nm). The field of view is illuminated by six halogen spots (20 W each), which are directly integrated into the camera housing (Figure 2). To attain accurate measurements, the ceiling lights, as well as other bright light sources, are switched off or covered during intraoperative or ex situ image recording. After processing spectral data, an RGB (red green blue) image and four false-color images are provided by the system. Each false-color image displays a quantitative analysis of physiologic parameters: tissue oxygenation (StO_2_), perfusion (NIR Perfusion Index (NIR-PI)), Tissue Water Index (TWI), and Tissue Hemoglobin Index (THI). While StO_2_ represents the relative blood oxygenation in the microcirculation of superficial tissue layers (penetration depth~1 mm), the NIR-PI represents tissue layers in 4–6 mm penetration depth. The indices THI and TWI display the distribution of hemoglobin and water in the observed tissue area, respectively. The parameters are specified in arbitrary units (a.u.) ranging from 0 to 100 (NIR-PI, TWI, THI) or expressed in percentages (StO_2_), which facilitate quantitative analyses and objective data comparison. A detailed description and validation of the described parameters are presented in the publications of Holmer et al. [31,44].

The main area of application of hyperspectral perfusion imaging in gastric cancer surgery is—in analogy to ICG-FI—the measurement of anastomotic perfusion. As both the esophagus and the small intestinal loop after Roux en Y pull-up are generally well perfused, we believe HSI to be of particular importance for subtotal oncologic gastrectomy, as it enables the evaluation of residual gastric stump perfusion and the performance of follow-up resection in cases of poor perfusion (Figure 1b,c).

However, owing to the paucity of available data, it is currently not possible to determine the respective limit and threshold values that might enable the differentiation between “ideal” and borderline perfusion values and their correlation with anastomotic healing. Furthermore, the large size of the currently available HSI camera system TIVITA^®^ Tissue has precluded the application of the system in both laparoscopic and robotic surgery in the recent past. A minimally invasive variant of the camera has recently been developed [45] and has been in clinical use in the University Hospital of Leipzig since 1 September 2021. This “TIVITA^®^ Mini”-camera for laparoscopic/thoracoscopic application promises to overcome the above mentioned limitations and respective studies with this new device have already been invented.

## 7. Hyperspectral Imaging in Gastric Cancer

Initial results of tissue-related diagnostics in gastric cancer using HSI are indicative of the huge potential of this technology with a view to endoscopic early diagnosis and intraoperative decisions on resection margins, safety distances, and extent of lymph node dissection, even though the histopathological examination still remains the gold standard.

**Figure 3:**
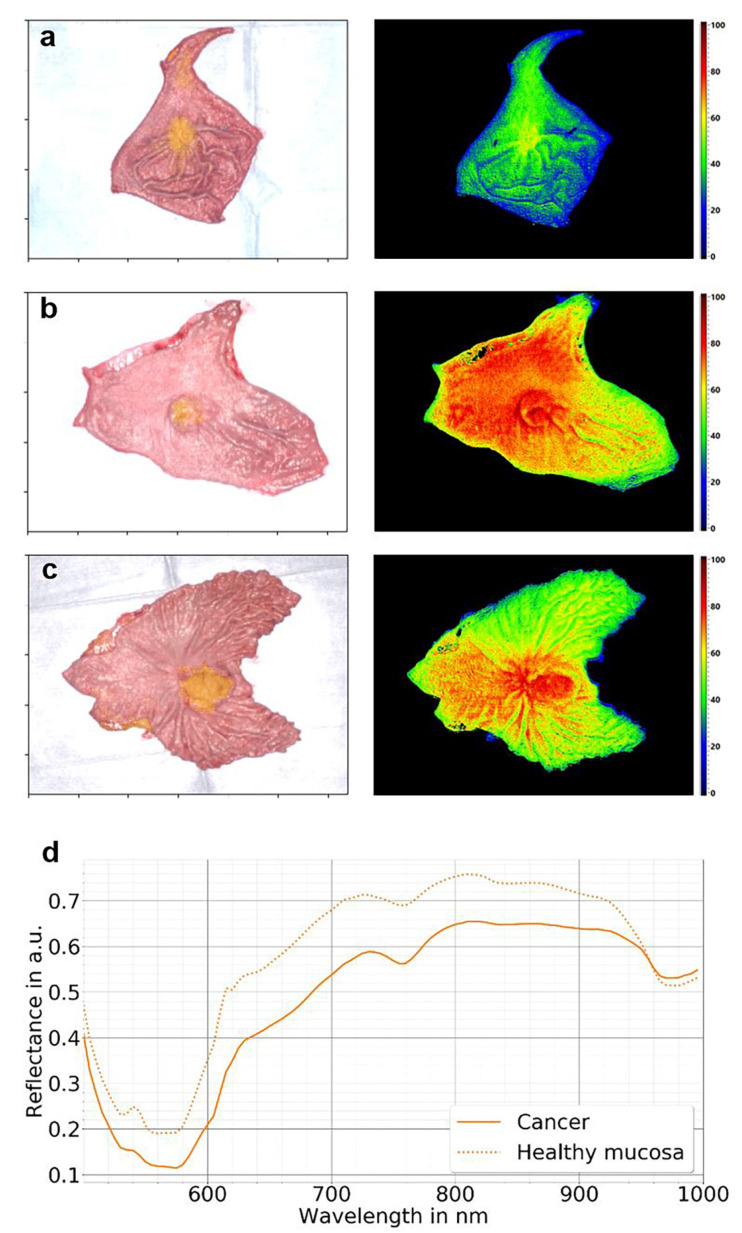
Visualization of HSI-based tissue classification in gastric cancer. Tissue classification was conducted in one case of early (**a**) and two cases of advanced gastric cancer (**b**,**c**). Hyperspectral images of the resected specimen were acquired immediately after resection and saline washout. The pictures on the right show intraoperatively computed false-color images of the tissue water index (TWI), as an example of visualized tissue characteristics. The tumor location was determined by visual assessment and histopathologic mapping procedures. Tissue classification was performed using a support vector machine. On the left, RGB images were superimposed with HSI-based classification results (yellow). The mean reflectance of the annotated cancerous tissue considerably differed from the findings in healthy mucosa (**d**).

In vivo and ex vivo tissue analyses using HSI techniques revealing the invisible features of cancer enable the capturing of significantly more information from a certain scene, within and beyond the visual spectral range (from 400 to 700 nm) [46]. It has thus become possible to differentiate and identify various structures, organs, and tissue types presented in a certain scene by their specific spectral signature (Figure 3).

First studies on gastric cancer detection using infrared HSI were published by Akbari et al. [23]. Results of their analyses led to groundbreaking advances in the optical diagnosis of cancer. Ten patients with gastric cancer were examined by HSI and the authors identified the appropriate wavelength region for cancer detection and spatially resolved images. Differences in the reflectance properties of cancerous versus non-cancerous tissues were highlighted and spectral signatures were extracted and evaluated in the different malignant and non-malignant tissue-types. First derivatives in the spectral regions and wavelengths ranging from 1226 to 1251 nm and 1288 to 1370 nm were proposed as criteria to successfully distinguish between non-cancerous and cancerous tissue.

Goto et al., have presented convincing evidence that HSI can be used to measure spectral reflectance (SR) in gastric tumors and to differentiate between tumorous and normal mucosa [47]. The authors examined the difference in the SR of pathological and normal gastric tissue. In 96 patients, a total of 104 gastric tumors removed by endoscopic submucosal dissection were examined. A wavelength of 770 nm and a cut-off value of 1/4 of the corrected SR were selected as the respective optimal wavelength and cut-off values. The rates of sensitivity, specificity, and accuracy of the diagnostic capabilities of the algorithm were 71%, 98%, and 85%, respectively. Tumor enhancement was achieved by image processing at 770 nm.

The results of recent publications suggest that the cutting-edge optical diagnostic technique NIR-HSI has the potential for gastric cancer diagnosis with suitable chemometrics. Recently, Liu et al., reported their findings for gastric cancer diagnosis using HSI with principal component analysis (PCA) and spectral angle mapper (SAM) [48]. PCA was applied to compress the dimension of hypercube data and to select the optimal wavelengths, whereas SAM was utilized for chemometrics to discriminate gastric cancer from normal tissue. The major spectral difference between cancerous and normal gastric tissue was observed at around 975 nm, 1215 nm, and 1450 nm. A total number of six wavelengths was defined as optimal by PCA. HE (Hematoxylin-Eosin) results showed an accuracy of up to 90% following the application of SAM.

**Figure 4:**
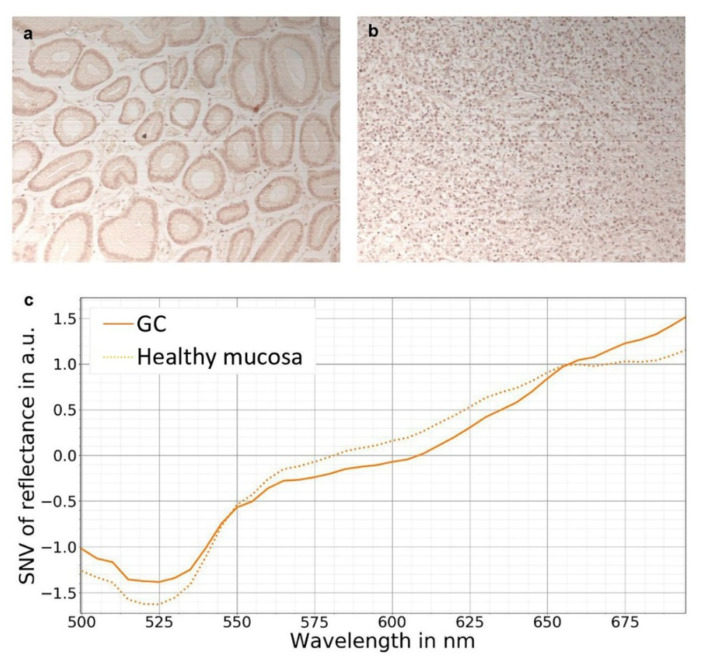
Exemplary determination of reflectance spectra in hematoxylin and eosin stained stomach specimens by HSI. Specimens from gastric cancer patients were stained with hematoxylin and eosin (HE). Areas with healthy gastric mucosa (**a**) and gastric cancer (GC) (**b**) were selected and imaged by a HSI camera as described previously [50]. The RGB images were reconstructed. The reflectance spectra (**c**) revealed distinct differences in the classes “healthy mucosa” and GC with the highest differences at the reflectance peaks of eosin (525 nm) and hematoxylin (630 nm).

HSI has further emerged into and significantly enriched the field of histopathologic diagnostics. Not only the detection of tumor cell nuclei, e.g., pancreatic tumors, has been shown via hyperspectral analysis of HE-stained pathological slides [49]; HSI-based gastric cancer detection has also become feasible with potential applications in computer-aided pathological diagnosis (Figure 4).

In addition, the combination of deep learning models and micro-spectral analyses has opened new perspectives for tissue characterization when compared to medical pathology [27,51]. Gastric tumor-classification based on micro-hyperspectral technology was performed by Hu et al. [52]. In their study, the authors established a hyperspectral database of 30 patients with gastric cancer. Based on the differences in spectral-spatial features between gastric cancer tissue and normal tissue in the spectral range of 410 to 910 nm, the authors proposed a deep-learning model-based analysis method for gastric cancer tissue. The analyses showed that the classification accuracy of the proposed model for cancerous and normal gastric tissue was 97.6% at a sensitivity and specificity of gastric cancer tissue of 97.2% and 98.0%, respectively. Interestingly, the method applied by the authors was able to fully extract the deep spectral-spatial features of tumor tissue.

The findings reported by Hu et al., are suggestive of the significant clinical impact and forward-looking possibilities of early gastric cancer detection based on deep learning combined with spectral imaging. However, available data on early gastric cancer are limited and only a small number of papers on the topic have been published so far. In the study by Li et al. [53], the fluorescence HSI technique was used to acquire fluorescence spectral images. Deep learning combined with spectral-spatial classification methods based on 120 fresh tissue samples with a diagnosis confirmed on histopathology was used to automatically identify and extract the “spectral + spatial” features to construct a model for the early diagnosis of gastric cancer. The results of the model showed that the overall accuracy for the non-precancerous lesion, precancerous lesion, and gastric cancer groups was 96.5% with specificities of 96.0%, 97.3%, and 96.7% and sensitivities of 97.0%, 96.3%, and 96.6%, respectively. The authors concluded that the use of the described method might increase the diagnostic accuracy and, thus, serve as a new tool for use in the early diagnosis of gastric cancer.

Current research focuses on exploring the value, reliability, and further development opportunities of HSI- or MSI-based tissue differentiation in clinical applications, such as multispectral in vivo endoscopy [54], and improving the automatic evaluation of the acquired data with the application of machine learning/artificial intelligence technologies [55]. Preliminary results are highly promising and future advancements in minimally invasive applications are of prime importance for extending utilization possibilities.
